# Modulation of oxidative phosphorylation and redox homeostasis in mitochondrial NDUFS4 deficiency via mesenchymal stem cells

**DOI:** 10.1186/s13287-017-0601-7

**Published:** 2017-06-24

**Authors:** Marlen Melcher, Katharina Danhauser, Annette Seibt, Özer Degistirici, Fabian Baertling, Arun Kumar Kondadi, Andreas S. Reichert, Werner J. H. Koopman, Peter H. G. M. Willems, Richard J. Rodenburg, Ertan Mayatepek, Roland Meisel, Felix Distelmaier

**Affiliations:** 1 Department of General Pediatrics, Neonatology and Pediatric Cardiology, University Children’s Hospital, Heinrich Heine University Düsseldorf, Moorenstraße 5, 40225 Düsseldorf, Germany; 2 Division of Pediatric Stem Cell Therapy, Clinic for Pediatric Oncology, Hematology and Clinical Immunology, Medical Faculty, Heinrich Heine University Medical Center, Düsseldorf, Germany; 3 Institute of Biochemistry and Molecular Biology I, Medical Faculty, Heinrich Heine University, Düsseldorf, Germany; 4 Department of Biochemistry (286), Radboud Institute for Molecular Life Sciences, Radboud University Nijmegen (Radboudumc), Nijmegen, The Netherlands; 5 Radboud Center for Mitochondrial Medicine (RCMM), Department of Pediatrics, 774 Translational Metabolic Laboratory (TML), Radboud University Medical Center, P.O. Box 9101, 6500 HB Nijmegen, The Netherlands

## Abstract

**Background:**

Disorders of the oxidative phosphorylation (OXPHOS) system represent a large group among the inborn errors of metabolism. The most frequently observed biochemical defect is isolated deficiency of mitochondrial complex I (CI). No effective treatment strategies for CI deficiency are so far available. The purpose of this study was to investigate whether and how mesenchymal stem cells (MSCs) are able to modulate metabolic function in fibroblast cell models of CI deficiency.

**Methods:**

We used human and murine fibroblasts with a defect in the nuclear DNA encoded NDUFS4 subunit of CI. Fibroblasts were co-cultured with MSCs under different stress conditions and intercellular mitochondrial transfer was assessed by flow cytometry and fluorescence microscopy. Reactive oxygen species (ROS) levels were measured using MitoSOX-Red. Protein levels of CI were analysed by blue native polyacrylamide gel electrophoresis (BN-PAGE).

**Results:**

Direct cellular interactions and mitochondrial transfer between MSCs and human as well as mouse fibroblast cell lines were demonstrated. Mitochondrial transfer was visible in 13.2% and 6% of fibroblasts (e.g. fibroblasts containing MSC mitochondria) for human and mouse cell lines, respectively. The transfer rate could be further stimulated via treatment of cells with TNF-α. MSCs effectively lowered cellular ROS production in NDUFS4-deficient fibroblast cell lines (either directly via co-culture or indirectly via incubation of cell lines with cell-free MSC supernatant). However, CI protein expression and activity were not rescued by MSC treatment.

**Conclusion:**

This study demonstrates the interplay between MSCs and fibroblast cell models of isolated CI deficiency including transfer of mitochondria as well as modulation of cellular ROS levels. Further exploration of these cellular interactions might help to develop MSC-based treatment strategies for human CI deficiency.

**Electronic supplementary material:**

The online version of this article (doi:10.1186/s13287-017-0601-7) contains supplementary material, which is available to authorized users.

## Background

Mitochondria are important cell organelles involved in many biological processes such as aerobic metabolism of glucose and fat, calcium signalling and apoptosis regulation [1–3]. Among the metabolic pathways located within mitochondria, oxidative phosphorylation (OXPHOS) plays a prominent role in cellular energy homeostasis. The system consists of four multi-protein complexes (CI–CIV) and the F_0_F_1_-ATP synthase (CV), embedded in the inner mitochondrial membrane [4, 5]. Disorders of the OXPHOS system can lead to a wide range of human diseases (e.g. Leigh disease, MELAS, LHON, MERRF, etc.), frequently affecting multiple organs. They can manifest at any age, with various modes of inheritance, and the number of genetically characterized OXPHOS diseases is constantly increasing [1, 6]. Mitochondrial CI (NADH:ubiquinone oxidoreductase) is the largest OXPHOS complex and constitutes one of the entry points for electrons into the electron transport chain. It consists of 44 different subunits, of which 37 are encoded by nuclear DNA (nDNA) and seven by mitochondrial DNA (mtDNA) [7, 8]. Among these subunits, the nuclear encoded NADH dehydrogenase ubiquinone Fe-S protein 4 (NDUFS4) is one of the most evolutionary conserved subunits, which is required for CI stability and function. Mutations within the *NDUFS4* gene are an important cause of early-onset Leigh syndrome [9–12]. Until now, treatment options for mitochondrial diseases are mainly supportive and no curative therapeutic approach is available for affected individuals.

Bone marrow-derived mesenchymal stem cells (MSCs) were discovered in 1966 [13] and 4 years later MSCs were isolated for the first time [14]. In the following years, based on their biological properties, MSCs emerged as a promising tool for cell-based treatment strategies for various human diseases—for example autoimmune disorders [15], osteopetrosis [16] and Alzheimer’s disease [17]. One prominent example for clinical application of MSCs is acute graft-versus-host disease (GVHD) during allogeneic stem cell transplantation [18]. The cellular effects of MSCs are partially related to immunosuppressive and immunomodulation effects on innate and adaptive immune cells and they escape recognition by alloreactive T cells [18, 19]. MSCs have been reported to exert beneficial effects on many other cell types. Several studies demonstrated that these effects are dependent on paracrine mechanisms, including secretion of microvesicles and exosomes [20, 21]. It was shown that MSCs have the ability of intercellular organelle transport (including mitochondria) via thin (diameter 50–200 nm) cytoplasmic bridges, named tunnelling nanotubes (TNTs), or via microvesicles [3, 22, 23]. The length of TNTs can be as long as the diameter of several cells. They contained F-actin, allowing a direct communication between distant cells. TNTs are dynamic structures that can be formed within a few minutes, but are fragile and highly sensitive to shearing forces and chemical fixatives [23, 24].

In the present study, we investigated the influence of MSCs on OXPHOS function in different cell culture systems of isolated CI deficiency caused by knockout or mutation of the *NDUFS4* gene. We demonstrate that MSCs can actively transport mitochondria to fibroblast cell lines. However, in human as well as mouse fibroblast cell lines this transfer was relatively low under resting conditions. Transfer efficiency was increased moderately by stimulation of the cells with TNF-α. The use of *OPA1* knockout cells, which have a defect in mitochondrial fusion, suggested that mitochondrial transfer between MSCs and fibroblasts per individual cell is underestimated in cell lines with normal mitochondrial dynamics. We show that although mitochondrial transfer was observed, reduced CI expression and function were not rescued. However, measurements of mitochondrial reactive oxygen species (ROS) levels indicated a positive effect of mitochondrial transfer on oxidative stress. Because the use of MSC-derived cell-free culture medium also mitigated ROS production, we conclude that MSCs display effects on cellular redox homeostasis independent of mitochondrial transfer.

## Methods

### Cells and culture conditions

We used MSCs of the mouse strain C57BL/6 (Life Technologies). The mouse mesenchymal stem cells (mMSCs) were plated on T75 flasks (Greiner bio-one) in Dulbecco’s Modified Eagle Medium/Nutrient Mixture F-12 (DMEM/F12; Gibco by life technologies) with GlutaMAX™-I (Gibco by life technologies) supplemented with 10% (v/v) fetal bovine serum (MSC FBS, qualified for mesenchymal stem cells; Gibco by life technologies) and 1% penicillin–streptomycin (5000 U/ml; Gibco by life technologies).

Human mesenchymal stem cells (hMSCs) were isolated as follows: 5–20 ml of heparinized human bone marrow aspirate was obtained from healthy volunteer donors after informed consent (this was approved by the local ethical committee of the Heinrich-Heine-University Düsseldorf; study number #1830). The collected sample were treated with red cell lysis buffer (155 mM ammonium chloride, 10 mM potassium hydrogencarbonate, 0.1 mM Na2EDTA · 2H_2_O; provided by the Hospital Pharmacy, University Clinic Düsseldorf, Germany) at a ratio of 1:2 and incubated for 10 min at room temperature. After washing with HBSS (Lonza), cells were re-suspended in complete medium consisting of Dulbecco’s Modified Eagle Medium low glucose (Lonza), 2 mM glutamin (Lonza), 1 IE/ml Na-heparin (Ratiopharm), 5% human fresh or frozen plasma and 5% platelet lysate (both provided by the Institute of Hemostasis and Transfusion Medicine, University Clinic Düsseldorf, Germany), and transferred at a density of ≤10^6^ WBC/cm^2^ into T175 flasks (Corning). Cells were cultured at 37 °C under 10% CO_2_ using a humidified incubator (Binder). Cells were allowed to adhere to the plastic surface for 24 h and then the medium was replaced. The removed supernatant containing non-adherent cells was transferred into new T175 flask and after further 24 h the supernatant with non-adherent cells was replaced by complete medium. When the cultures reached approximately 70–80% confluence, adherent cells were detached by treatment with TrypLE (Invitrogen) and re-plated into tissue flasks (all from Corning) for further passages. For experiments, hMSCs of passage 1–3 were used. The multipotentiality of the MSCs was verified with the use of in-vitro assays to differentiate MSCs into adipocytes (StemPro Adipogenesis Differentiation Kit; Gibco) and chondrocytes (type II collagen staining according to the protocol of PromoCell).

Primary human skin fibroblasts (NHDF-neo, Lot Number 7 F3956, 0000476623 and 0000478986; Lonza) and wild-type (WT) mouse fibroblasts [9] were used as control cell lines in the experiments described in the following.

Cell culture model systems of NDUFS4 deficiency included an established immortalized embryonic mouse fibroblast cell line [9], which has a knockout of *NDUFS4* (*NDUFS4* KO) [1], and a primary human skin fibroblast cell line from a patient with a homozygous nonsense mutation c.20C > G; p.(Ser7*) in *NDUFS4* (NM_002495.2). The use of these cell lines for this study was approved by the local ethical committee of the Heinrich-Heine-University Düsseldorf (study number #4272). In patient fibroblasts, NDUFS4 protein was undetectable and CI activity was drastically reduced (see Additional file 1).

For additional control experiments, we obtained immortalized embryonic mouse fibroblasts from the Institute of Biochemistry and Molecular Biology I (Medical Faculty, Heinrich-Heine-University Düsseldorf), which have a knockout of the mitochondrial inner membrane fusion protein optic atrophy 1 (*OPA1* KO). All fibroblasts cell lines were cultured in DMEM with GlutaMAX™-I (Gibco by life technologies) supplemented with 10% FBS and 1% penicillin–streptomycin (5000 U/ml). After fluorescence labelling via transduction, all cell lines were cultured in selection medium (3 μg/ml blasticidin; Gibco by life technologies). The medium was replaced every 2–3 days, cells were split when the desired confluence (70–90%) had been reached and the experiments started at the earliest 2 weeks after transduction.

### Co-culture experiments

Fibroblasts and MSCs from the same species were co-cultured in stem cell medium without blasticidin. For drug treatment experiments, cells were co-cultured on six-well plates (9.6 cm^2^ per well; Greiner CELLSTAR﻿). For ROS measurements and fluorescence microscopy, cells were co-cultured on glass-bottom dishes (27 mm; Nunc by Thermo Fisher Scientific). Stem cells and fibroblasts were co-cultured in a ratio of 2:1 (drug treatment experiments) and in a ratio of 1:1 (ROS measurements and fluorescence microscopy) for 72 h in a humidified atmosphere (95% air, 5% CO_2_) at 37 °C.

### Drug treatment

Galactose (Gal), 2-deoxy-d-glucose (2DG), d,l-buthionine-(*S*, *R*)-sulfoximine (BSO) and tumour necrosis factor alpha (TNF-α) were obtained from Sigma-Aldrich. The fibroblasts were pre-treated for 72 h (Gal) or 24 h (BSO, 2DG). DMEM without Glucose (Gibco by life technologies) supplemented with 10% FBS and 1% penicillin–streptomycin (5,000U/ml) was used for the drug treatment experiments. TNF-α was added to the cells during the co-culture. The following concentrations were used: 5.5 mM Gal, 20 mM 2DG, 1 mM and 100 μM BSO, and 0.5, 1.0, 1.5 and 15 μg/ml TNF-α.

### SDS-PAGE and western blot analysis

First, mitochondria were isolated as described previously [25] and the protein concentration was determined (Pierce™ BCA Protein Assay Kit; ThermoFisher scientific). Then, proteins were separated by sodium dodecyl sulphate polyacrylamide gel electrophoresis (SDS-PAGE) on 4–12% protein gels (NuPAGE™ Novex™ 4–12% Bis–Tris protein gels; Invitrogen). Gels were transferred to 0.2 μm nitrocellulose membrane (1 h at 200 mA; Bio-Rad). Membranes were blocked and incubated with primary antibodies (anti-NDUFS4 (Sigma-Aldrich), polyclonal, 1:1000; voltage-dependent anion channel (VDAC; Cell Signaling), monoclonal, 1:1000; or anti-SDHA (Mitosciences MS204), 1:1000) overnight at 4 °C. Horseradish peroxidase-linked anti-mouse IgG antibody (1:1000; GE Healthcare) or anti-rabbit IgG (1:1000; GE Healthcare) were used as secondary antibodies.

### Preparation of MSC supernatants

The MSC supernatant preparation was performed with some modifications as described previously [21]. Briefly, medium (supernatant) from MSCs was collected and centrifuged at 300 × *g* for 10 min to remove the remaining cells. Subsequently, the supernatant was transferred to a new tube and centrifuged at 2000 × *g* for 10 min. The cell and cell debris-free supernatant was then added to the fibroblasts. Every 24 h, fresh cell-free MSC supernatant was added to the fibroblasts over a period of 72 h.

### Fluorescent labelling

The sequences of the organelle markers Cox8a (Cytochrome c oxidase subunit 8A) GFP, Cox8a RFP, LMNB (Lamin B1) RFP and LMNB BFP were amplified from pENTR plasmids from OriGene. A start and stop codon was added by polymerase chain reaction (PCR). Open reading frames of organelle markers were subcloned in pLenti6.3/V5-TOPO Vector using the pLenti6.3/V5-TOPO TA Cloning Kit (Invitrogen). Stable cell lines from hMSCs and mMSCs with green fluorescent mitochondria (Cox8a GFP) and from human and mouse fibroblasts with red fluorescent mitochondria (Cox8a RFP) and red or blue fluorescence nucleus (LMNB RFP or LMNB BFP) were produced by lentiviral transduction. Transduction was performed according to the manufacturer’s protocol with some modifications. Briefly, HEK-293 T cells (HEK-T) were seeded in T75 flasks (1 × 10^6^ cells/ flask) in DMEM with 10% FBS. After 24–48 h the HEK-T had reached a confluence of 70% and the medium was changed to 5 ml OptiMEM (Gibco by life technologies). A mixture of the plasmids (pLenti6.3/V5 + insert and packing plasmids) and Lipofectamin 2000 (Invitrogen) in OptiMEM was added to the HEK-T. The medium was replaced after 24 h by DMEM with 10% FBS passed through a 0.45-μM Millex-HV filter (Merck Millipore). Again 24 h later, the supernatant from the HEK-T was added to the cells to be transduced (mMSCs, MSCs, fibroblasts). Using a high-speed sorter (MoFlo XDP; Beckman-Coulter), the cells were highly enriched (RFP, GFP or BFP positive) 2 weeks after transduction.

### Fluorescence imaging of mitochondrial transfer

After co-culturing, cells were stained with phalloidin (mouse cells with Alexa Fluor 594, human cells with Alexa Fluor 650 phalloidin; Molecular Probes). First, cells were fixed for 20 min in the dark with formalin, washed with PBS and permeabilized with 0.3% triton in PBS. Then, cells were incubated for 20 min with phalloidin. Images were obtained by fluorescence microscopy (40× objective; Zeiss; Axio Observer Z1 microscope with ApoTome 2 and a Perkin-Elmer Ultra View spinning disc confocal microscope) equipped with a GFP filterset (excitation: 520/28 BrightLine HC, emission: 482/18 BrightLine HC; AHF Analysetechnik), DAPI HC BrightLine Basic filterset BFP (excitation: 390/18 BrightLine HC, emission: 460/60 BrightLine HC; AHF Analysetechnik), Filterset 31 (excitation: BP 565/30, emission: BP 620/60; Zeiss) and Filterset 50 (excitation: BP 640/30, emission: BP 690/50; Zeiss). For live cell microscopy with recording of time-lapse videos (see Additional files 4 and 5), images were acquired at intervals of 30 sec for a duration of 8 min.

### Measurements of ROS levels

The measurements of cellular ROS production were performed with some modifications as described previously [5]. Briefly, after co-culture for 72 h, cells were incubated with MSC medium containing MitoSOX Red (5 μM, 10 min, 37 ° C; Invitrogen). The red fluorescence was documented using an Axio Observer Z1 microscope (Zeiss) with the dehydroethidium filterset (F39-500, F48-515, F47-895; AHF Analysetechnik). Images were analysed and fluorescence intensity was quantified using ImageJ software (Wayne Rasband at the National Institutes of Health; http://rsbweb.nih.gov/ij/).

### Quantification of mitochondrial mass

Mitochondrial mass was determined as described previously with some modifications [26]. We used Cox8a-labelled fibroblasts and Cox8a-labelled MSCs for this experiment.

### Analysis of mitochondrial transfer by flow cytometry

After co-culture (24, 48 or 72 h), cells were detached with TrypLE select (Gibco by life technologies), washed with PBS and fixed with Fixation Buffer (BioLegend) for 20 min in the dark. Subsequently the probes were washed again with PBS and finally re-suspended in 100 μl PBS. A FACS Canto-II flow cytometer (BD Bioscience) was used for the measurements and data analysis was performed using FlowJo software version 10. Fibroblasts and stem cells were distinguished by their different fluorescence labelling. Only the mitochondria transfer from stem cells to fibroblasts was quantified. Approximately 30,000 cells were evaluated for each sample.

### Blue native polyacrylamide gel electrophoresis

Blue native polyacrylamide gel electrophoresis (BN-PAGE) was performed according to a previously described standard protocol [27] with slight modifications. In brief, mitochondria-enriched cell fractions were generated by re-suspending cell pellets in 2% digitonin followed by centrifugation. These fractions were re-suspended in 2% *n*-dodecyl β-d-maltoside to solubilize membrane proteins. Then 100 μg of mitochondrial protein was loaded for each sample and separated on a 4–12% BN-PAGE gradient gel. After BN-PAGE, gels were subjected to western blotting and immunodetection as already described. Complex I in-gel-activity assay was performed by incubating the blue native gel with complex I IGA solution (3 mM nitrotetrazolium blue, 150 μM NADH, 2 mM Tris–HCl, pH 7.4) for 2 h at room temperature immediately after the gel run. After briefly washing the gel with ultrapure water, images of the gel were captured using a gel scanner (Image Scanner III; GE Healthcare).

### Measurement of complex I enzyme activity

Cells were cultured for 72 h with or without supernatant from MSCs. Activity of complex I was measured using a complex I enzyme activity microplate assay kit (Abcam) according to the manufacturer’s protocol.

### Statistical analysis

Unless otherwise described, experimental data are shown as mean ± standard error of the mean (SEM) or standard deviation and the statistical significance between datasets is assessed using an independent two-population Student’s *t* test. *p* < 0.05 was considered significant. All statistical analysis was performed using GraphPad Prism 7.

## Results

### MSCs transfer mitochondria to fibroblasts with NDUFS4 deficiency

Mitochondrial transfer via TNTs in vitro has already been described for some cell types—for example between endothelial cells and cancer cells [28], between rat renal tubular cells and MSCs [29] and between cardiomyocytes and MSCs [22]. However, this phenomenon was not analysed in nuclear DNA encoded CI defects. Moreover, studies suggest that mitochondrial transfer might only be relevant in cell models with near total absence of mitochondrial function and does not play a role in human diseases caused by mitochondrial dysfunction [30].

**Fig. 1:**
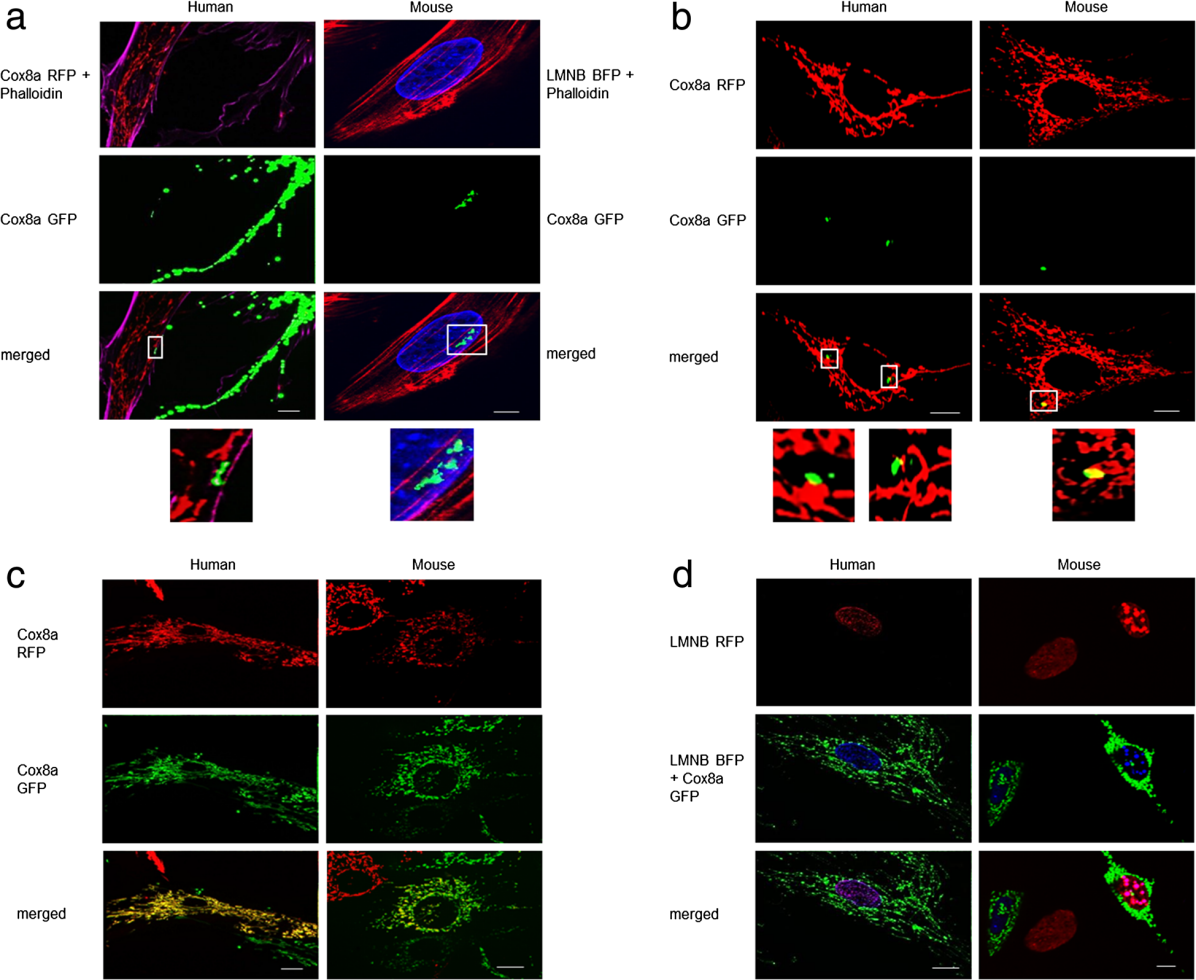
**a**, **b** Mitochondrial transfer from MSCs to fibroblast cell lines. **a** Representative fluorescence images showing mitochondrial transfer and TNT formation. Mitochondria of stem cells are labelled with Cox8a GFP (green fluorescent protein), mitochondria of human fibroblasts are labelled with Cox8a RFP (red fluorescent protein) and nuclei of murine fibroblasts are labelled with LMNB BFP (blue fluorescent protein). Co-cultures were fixed and stained with phalloidin. **b** Confocal images of mitochondrial transfer derived from MSCs. Fibroblast mitochondria are labelled with Cox8a RFP and stem cell mitochondria are labelled with Cox8a GFP. **c**, **d** Representative fluorescence images of cell fusion between stem cell and fibroblast. **c** Mitochondria are labelled with Cox8a GFP (stem cell) and Cox8a RFP (fibroblast). Cells with a complete co-localization of red and green fluorescence indicate the rare event of nuclear fusion (yellow cells). **d** Stem cells have Cox8a GFP-labelled mitochondria and LMNB BFP-labelled nuclei. Fibroblasts have LMNB RFP-labelled nuclei. Cells with a co-localization of LMNB BFP and LMNB RFP have fused nuclei. *Scale bars* represent 10 μm. Images were contrast optimized (Colour figure online). Cox8a Cytochrome c oxidase subunit 8A, LMNB Lamin B1

To investigate whether MSCs are able to transfer mitochondria in two model systems of mitochondrial NDUFS4 deficiency (e.g. patient-derived fibroblasts with pathogenic *NDUFS4* mutations and mouse fibroblast with *NDUFS4* knockout), we performed co-culture studies of MSCs with fibroblast cell lines. For visualization, MSC mitochondria were labelled with Cox8a GFP and fibroblast mitochondria with Cox8a RFP or the nuclei with LMNB BFP. After co-culture, cell boundaries were additionally stained with phalloidin, which binds to actin filaments. Next, cell cultures were examined by fluorescence microscopy. Video imaging studies of living cells as well as confocal microscopy studies indicated the formation of thin cytoplasmic bridges between the different cell lines, which most likely represent actin filament-based TNTs. Moreover, for human and mouse cell lines, we observed transfer of mitochondria between MSCs and fibroblasts (Fig. 1a, b; see also Additional files 2 and 3 as well as the time-lapse videos in Additional files 4 and 5). Of note, this appeared not to be a unidirectional transfer but also occurred from fibroblasts to MSCs.

In addition to the transfer of isolated/small amounts of mitochondria we also identified cells with complete co-localization of MSC and fibroblast mitochondria (e.g. a yellow mitochondrial network; Fig. 1c). These findings suggested cellular or even nuclear fusion. To further investigate the phenomenon, we labelled MSC nuclei with LMNB BFP and mitochondria with Cox8a GFP and the fibroblast nuclei with LMNB RFP. As depicted in Fig. 1d, we observed a complete co-localization of MSC and fibroblast nuclei (Fig. 1d). This suggests complete cellular and nuclear fusion. However, at less than 0.1%, the complete cell fusion appeared to be an extremely rare phenomenon (data not shown).

### TNF-α increases the efficiency of mitochondrial transfer

**Fig. 2:**
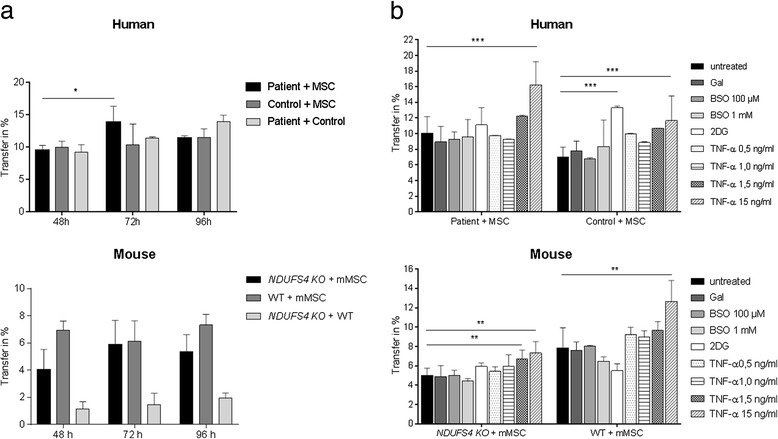
Quantitative FACS measurement of mitochondrial transfer. **a** Flow cytometry analysis was performed after 48, 72 and 96 h of co-culture. Co-cultures between MSCs and fibroblasts and between fibroblasts and fibroblasts (e.g. healthy controls with NDUFS4-deficient cell lines) were measured. **b** Mitochondrial transfer during different culture conditions/drug treatment. *mMSC* mouse mesenchymal stem cell, *MSC* mesenchymal stem cell, *Gal* galactose medium, *BSO* buthioninesulfoximine, *2DG* 2-deoxy-d-glucose, *TNF-α* tumour necrosis factor alpha, *NDUFS4* NADH dehydrogenase ubiquinone Fe-S protein 4, *WT* wild type. Data shown as mean (% of “mito-positive” cells per measurement) ± SD. *p < 0.05, **p < 0.01, ***p < 0.001

The amount of mitochondrial transfer was determined by flow cytometry using LMNB RFP-labelled fibroblasts and Cox8a GFP-labelled MSCs. After co-cultivation, a subpopulation was observed, which was LMNB RFP and Cox8a GFP positive. Next, we determined after which time period (48, 72 or 96 h) the transfer rate between patient and hMSCs or between NDUFS4 knockout and mMSCs was most efficient (for illustration of the method used, see also Additional file 3). As depicted in Fig. 2a, transfer appeared optimal after 72 h (human 13.2% and mouse 6%). For this reason, we chose for all further co-culture experiments a time period of 72 h. Of note, we observed slight differences between the human and the murine model systems. For example, in the murine system mitochondrial transfer between NDUFS4 knockout cells and control fibroblasts was rather low (1–2%), but in the human system the transfer between patient and control fibroblasts is almost as frequent as between patient fibroblasts and MSCs.

As shown in Fig. 2a, transfer efficiency in both model systems was relatively low. However, low transfer rate might be influenced by cell culture conditions (e.g. standard fibroblast culture provides optimal metabolic supply, which potentially masks the OXPHOS defect). Therefore, our hypothesis was that metabolic or oxidative stress might increase mitochondrial transfer.

To stimulate transfer, fibroblasts were subjected to stress condition by culturing in galactose medium as well as BSO and 2DG treatment (Fig. 2b). Galactose is a frequently used metabolic stress treatment, increasing the dependence on OXPHOS-linked ATP production [31]. BSO inhibits the glutamate cysteine ligase, which is the rate limiting factor for glutathione synthesis. Glutathione is an important cellular antioxidant. By inhibition of the glutamate cysteine ligase, cells are exposed to oxidative stress [32]. 2DG is a glucose analogue that partially inhibits glycolysis, thereby reducing glycolytic flux. This inhibition occurs by its action on hexokinase, which is the rate limiting step of glycolysis [33].

As depicted in Fig. 2b, culturing in galactose medium and BSO treatment did not alter transfer efficiency in both model systems. Interestingly, 2DG treatment moderately increased mitochondrial transfer between human control fibroblasts and hMSCs.

Next, we evaluated the effects of TNF-α treatment in the different cell lines. TNF-α is known to stimulate the formation of TNTs by remodelling of actin filaments [34]. As shown in Fig. 2b, TNF-α dose-dependently stimulated transfer efficiency with significant effects at a relatively high concentration of 15 ng/ml between human patient fibroblasts and hMSCs (10.1% ± 2.1 without TNF-α and 16.3% ± 2.9 with 15 ng/ml TNF-α) and between NDUFS4 KO and mMSCs (5% ± 0.8 without TNF-α and 7.4 ± 1.2 with 15 ng/ml TNF-α).

### Abrogating mitochondrial fusion indicates transfer of more mitochondrial mass per individual cell

**Fig. 3:**
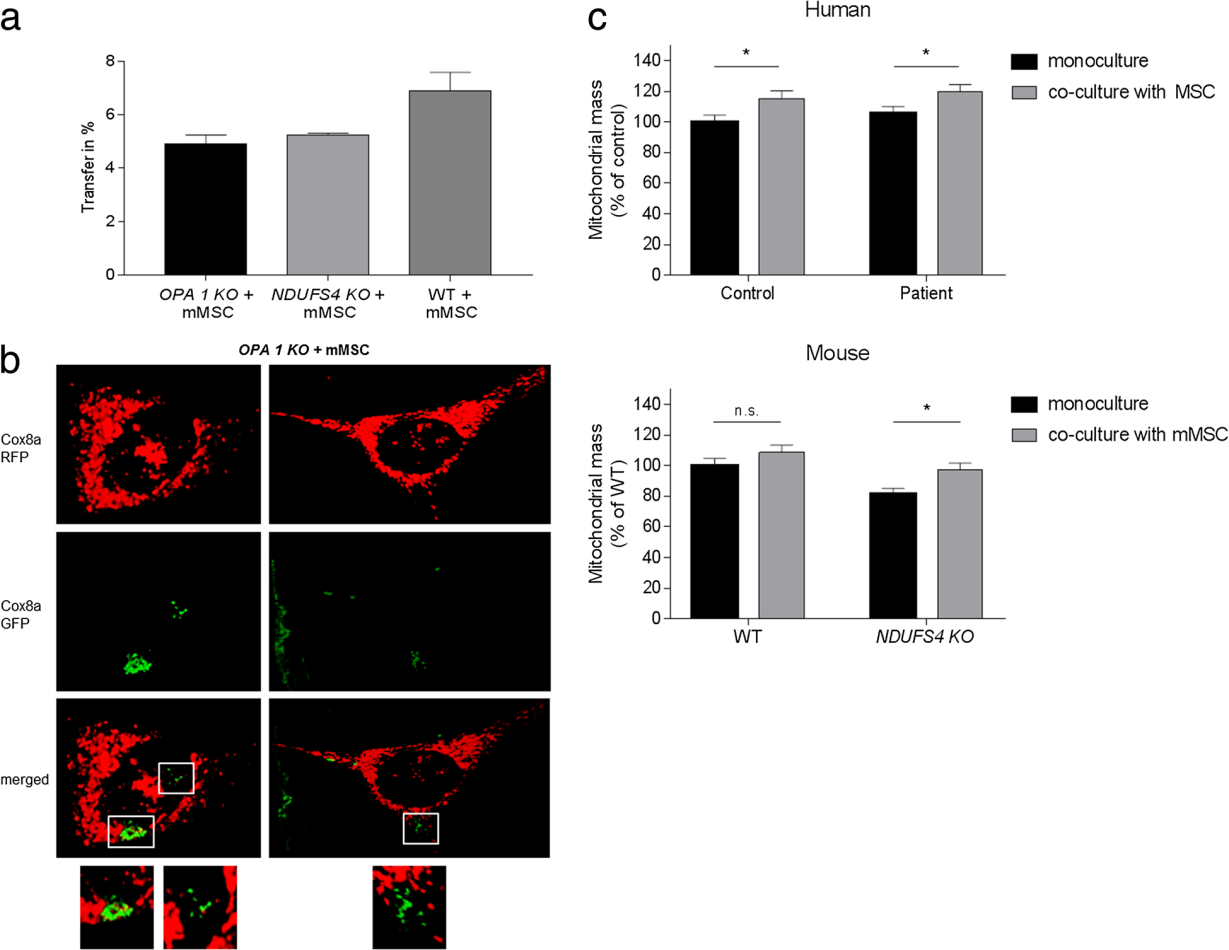
Mitochondrial transfer between OPA1 KO fibroblasts and murine MSCs. **a** OPA1 KO fibroblasts were transfected with LMNB RFP (red fluorescent protein) to label the nuclei and mMSCs were transfected with Cox8a GFP (green fluorescent protein) to label the mitochondria. Flow cytometry was performed after 72 h co-culture. Data shown as mean (% of “mito-positive” cells per measurement) ± standard deviation. **b** Fluorescence images of mitochondrial transfer. Mitochondria of OPA1 KO fibroblasts were transfected with Cox8a RFP and stem cell mitochondria were transfected with Cox8a GFP. *Scale bars* represent 10 μm. Images were contrast optimized. **c** Mitochondrial mass was measured via live-cell microscopy in fibroblasts with and without visible mitochondrial transfer from MSCs. A significant increase was detected in fibroblasts that received MSC mitochondria during co-cultivation. *p < 0.05. *n.s.* not significant, *mMSC* mouse mesenchymal stem cell, *MSC* mesenchymal stem cell, *NDUFS4* NADH dehydrogenase ubiquinone Fe-S protein 4, *OPA1* optic atrophy 1, *WT* wild type (Colour figure online)

Even during the aforementioned TNF-α treatment, the efficiency of mitochondrial transfer appeared to be relatively low. However, mitochondria are dynamic organelles, which constantly go through the fission/fusion cycle. Therefore, it might be possible that transferred mitochondria rapidly fuse with the mitochondrial network of the “host cell” so that the fluorescence signal is diluted and thereby the transfer rate could be underestimated. For this reason, mMSCs were co-cultured with a murine *OPA1* KO cell line. The OPA1 protein is essential for mitochondrial fusion and cells without functional OPA1 have a fragmented mitochondrial network that is unable to incorporate/fuse mitochondria [35]. For the experiments depicted in Fig. 3a, the nuclei of the *OPA1* KO fibroblasts were labelled with LMNB RFP and the mMSC mitochondria were labelled with Cox8a GFP. Transfer was analysed by flow cytometry after co-cultivation for 72 h. A higher transfer rate between *OPA1* KO fibroblast and mMSCs could not be detected (e.g. no increase in the double-stained cell population; Fig. 3a). To investigate the mitochondrial transfer on the single cell level, *OPA1* KO mitochondria were labelled with Cox8a RFP and mMSC mitochondria with Cox8a GFP. Using confocal microscopy, we observed that, despite the earlier findings, larger amounts of mMSC mitochondria were transferred to *OPA1* KO fibroblasts per single cell (Fig. 3b). This suggests that the images depicted in Fig. 1 potentially under-represent mitochondrial transfer because of incorporation into the mitochondrial network of the acceptor cell or because of degradation via autophagy/mitophagy. Interestingly, analysis of the mitochondrial mass in human and mouse NDUFS4-deficient fibroblasts revealed that cells with uptake of MSC mitochondria had a significant increase in mitochondrial mass (Fig. 3c). This argues against the idea that only single mitochondria are transferred and speaks against the hypothesis that transferred mitochondria are rapidly degraded via autophagy.

### MSCs exert antioxidative effects on NDUFS4-deficient fibroblasts

Mitochondria are a major source of cellular ROS production via the process of OXPHOS (in particular via CI and CIII) [36]. This phenomenon might be aggravated under conditions of OXPHOS dysfunction. In fibroblasts of patients with nuclear encoded CI deficiency it was shown that superoxide production as well as hydrogen peroxide levels are significantly elevated [37].

Using the fluorescent probe MitoSOX Red, measurements of mitochondrial ROS levels were carried out via live-cell microscopy. For this analysis, stem cell mitochondria were labelled with Cox8a GFP and the nuclei of NDUFS4-deficient fibroblasts were labelled with LMNB BFP. Using this approach, we were able to measure NDUFS4-deficient fibroblasts with and without mitochondrial transfer on the same coverslip.

**Fig. 4:**
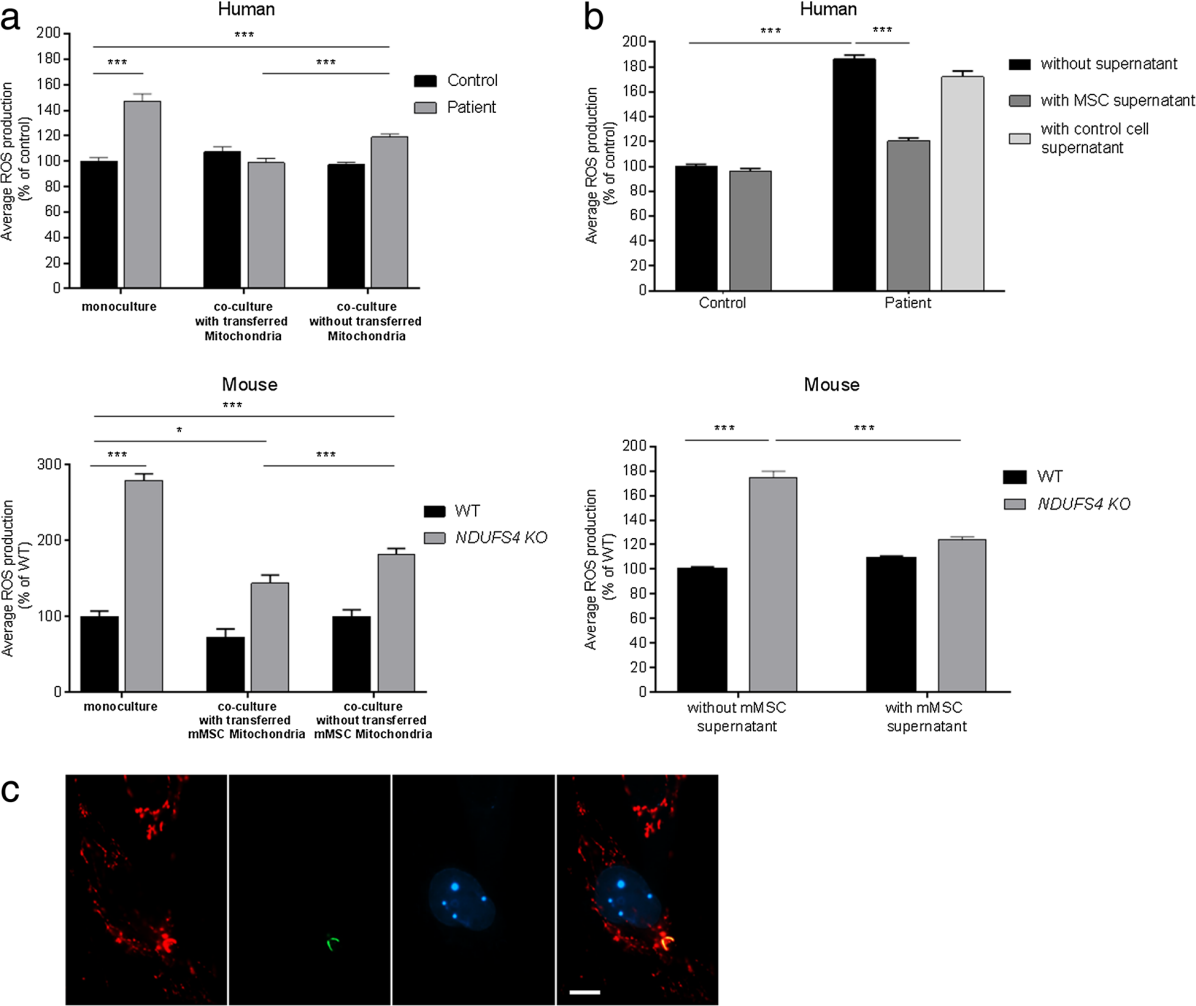
Mitochondrial ROS production was measured using MitoSOX Red (mitochondria-targeted superoxide indicator). **a** Fibroblast nuclei were labelled with LMNB BFP (blue fluorescent protein) and stem cell mitochondria were labelled with Cox8a GFP. Only fibroblasts with a blue fluorescent nucleus were measured. The measured fibroblasts were divided into two groups: fibroblasts with obviously transferred mitochondria derived from MSCs; and fibroblasts without transferred mitochondria. **b** MitoSOX measurement was performed after 72-h treatment with stem cell supernatant or control cell supernatant. Data shown as mean ± SEM. *p < 0.05, ***p < 0.001. **c** Representative fluorescence images showing a transferred MSC mitochondrion (*green*) in a human fibroblast (*blue nuclei*). Mitochondrial MitoSOX fluorescence is shown (*red*). *Scale bar* represents 10 μm. *mMSC* mouse mesenchymal stem cell, *MSC* mesenchymal stem cell, *NDUFS4* NADH dehydrogenase ubiquinone Fe-S protein 4, *ROS* reactive oxygen species, *WT* wild type (Colour figure online)

Results in human cell lines demonstrated increased ROS levels in NDUFS4-deficient fibroblasts (Fig. 4a, upper panel). After co-cultivation of human fibroblasts with hMSCs, the ROS levels in patient fibroblasts that had received hMSC mitochondria normalized. Figure 4c shows a representative fibroblast with transferred mitochondria from MSCs (green) and the MitoSOX staining (red). However, we also observed that patient fibroblasts without obvious mitochondrial transfer showed lower ROS levels compared with untreated conditions. This finding suggests that apart from direct mitochondrial transfer, additional factors (e.g. paracrine factors, microvesicles, etc.) might play a role during co-culture conditions.

Similar findings were obtained when studying the mouse cell model system (Fig. 4a, lower panel). Of note, ROS levels in mouse *NDUFS4* KO cells were only elevated under low-glucose culture conditions and normalized under high-glucose conditions, which suggests differences in cell metabolism compared with primary human cells.

To investigate the effects of MSCs independent of mitochondrial transfer, we cultured fibroblasts with stem cell medium supernatant, which was free of stem cells and cell debris. To this end, every 24 h the supernatant was replaced by fresh supernatant and after 72 h we measured ROS levels using MitoSOX Red. Fibroblasts treated with stem cell supernatant produced significantly less ROS compared with untreated fibroblasts (Fig. 4b). This supports the earlier idea that MSC-derived factors are released into the cell culture medium to modulate biological functions of other cell types.

### Mitochondrial transfer and mitigation of cellular ROS production are unable to rescue CI deficiency in NDUFS4-deficient fibroblasts

**Fig. 5:**
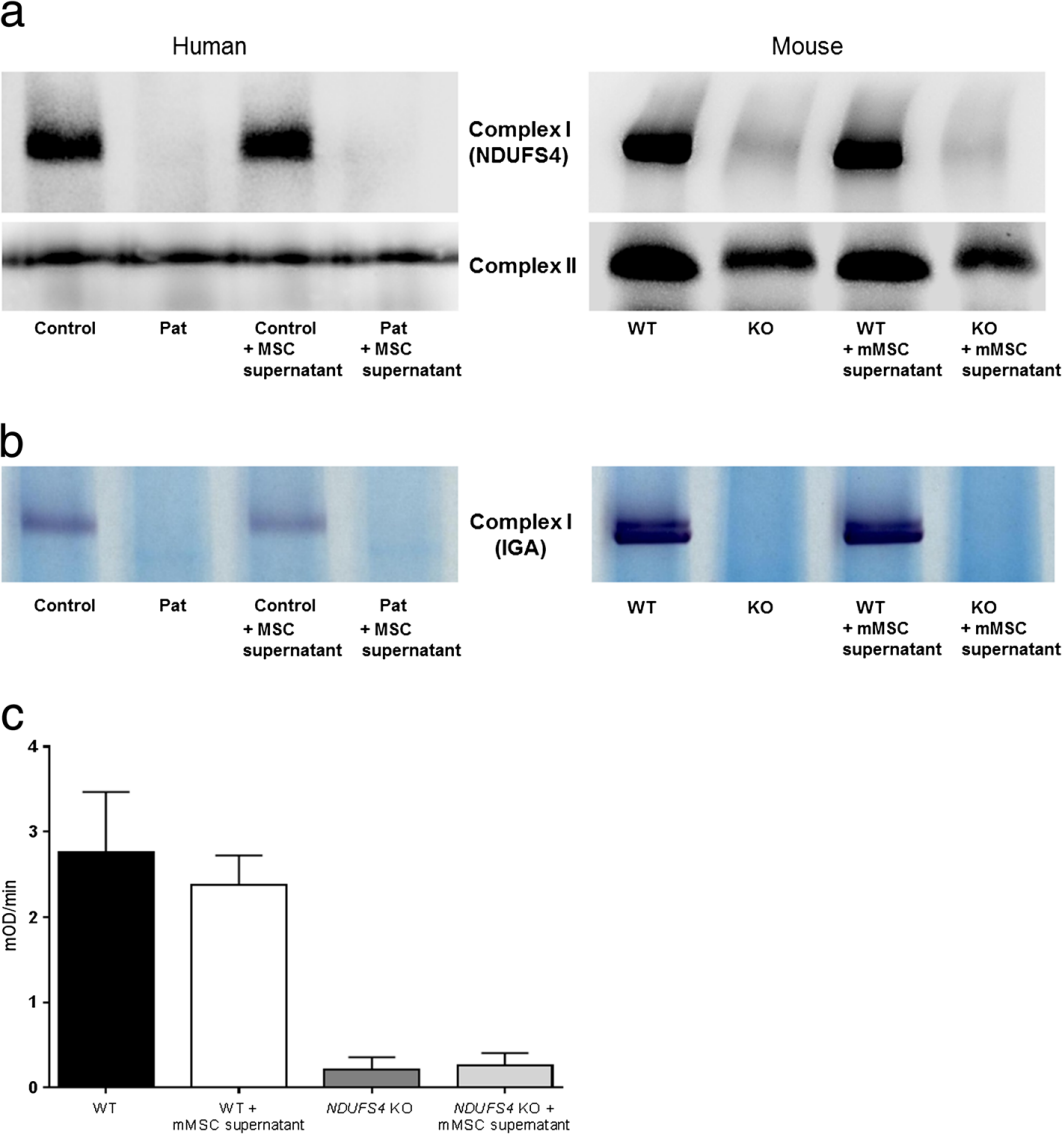
**a** BN-PAGE with mitochondrial lysates from human and mouse fibroblasts cell lines shows no recovery of fully assembled complex I levels upon treatment with MSC supernatant. Complex II SDHA was used as loading control. **b** Complex I in-gel-activity assay demonstrates no improvement of CI activity upon treatment with MSC supernatant. **c** A complex I enzyme activity microplate assay kit was used to determine the complex I activity in mouse *NDUFS4* KO and WT fibroblasts treated or untreated with supernatant from mMSCs. In accordance with BN-PAGE analysis, no significant increase in activity could be detected. Rate (mOD/min) = Absorbance 1 – Absorbance 2 / time (min). *mMSC* mouse mesenchymal stem cell, *MSC* mesenchymal stem cell, *NDUFS4* NADH dehydrogenase ubiquinone Fe-S protein 4, *WT* wild type

The NDUFS4 subunit was not detectable in patient fibroblasts and in murine *NDUFS4* KO fibroblasts (see Additional file 1A). CI activity of NDUFS4-deficient patient fibroblasts was less than 10% of the average mean in control cells. Without the NDUFS4 subunit CI is very unstable, which results in a reduced activity in NDUFS4-deficient fibroblasts [38]. To check whether treatment of fibroblasts with MSC supernatant leads to an increase of fully assembled CI, BN-PAGE was performed. Even after 72 h of treatment no assembled CI or CI in-gel activity could be detected in NDUFS4-deficient fibroblasts, neither in the mouse nor the human system (Fig. 5). Similar findings were obtained using a complex I enzyme activity microplate assay in mouse fibroblasts (Fig. 5c). Secreted factors of MSCs are not sufficient to increase CI levels of the NDUFS4-deficient fibroblasts to a detectable level.

## Discussion

Intercellular communication is an essential process for the development and maintenance of multicellular organisms. The discovery of nanotube-mediated membrane continuity between mammalian cells triggered a renewed interest in cell–cell interactions [39]. Several studies indicate that MSCs can affect the aerobic respiration of neighbouring cells by TNT-mediated mitochondrial transfer [3]. The current study aimed to determine whether mitochondrial transfer occurred from MSCs to fibroblasts with mitochondrial dysfunction due to *NDUFS4* mutation (human cells) or knockout (mouse cells).

We demonstrate that, under physiological conditions, mitochondria are transferred from MSCs to fibroblasts albeit at a relatively low efficiency (6–14%). Evidence from the literature suggested that mitochondrial transfer is increased by conditions of oxidative stress, during conditions of metabolic stress and by TNF-α treatment. Zhu et al. [40] analysed the effects of oxidative stress on the F-actin fibres in astrocytes. They found that increasing ROS levels via exogenous applications of H_2_O_2_ stimulates the polymerization of actin and thus the formation of TNT-like structures. Liu et al. [41] reported a mitochondrial transfer rate of approximately 40% between MSCs and human umbilical vein endothelial cells after oxygen glucose deprivation and subsequent reoxygenation (under normal conditions 8%). Ahmad et al. [42] showed an increased transfer rate between mouse lung epithelial cells (LA4) and stem cells after stress treatment with Rotenone.

Our model involving application of BSO, a substance that reduces the glutathione pool and thereby raises ROS levels, did not detectably increase mitochondrial transfer. Similarly, induction of metabolic stress by glycolysis inhibition using 2DG or GAL treatment exerted only minor (2DG) or no (GAL) effect on mitochondrial transfer. Only TNF-α treatment dose-dependently stimulated transfer efficiency with significant effects at a relatively high concentration. This observation suggests that MSCs respond particularly well to immunological stimuli. MSCs show immune homeostatic functions, which are enhanced by the exposure of pro-inflammatory cytokines, such as TNF-α [43]. High concentrations of TNF-α trigger the F-actin polymerization and thereby the formation of TNTs. This phenomenon most likely contributes to increased mitochondrial transfer from MSCs to fibroblasts [44].

Of note, determining mitochondrial transfer is challenging because mitochondria are constantly involved in the processes of fission and fusion. Mitochondrial fusion can be stimulated by oxidative stress and defective mitochondria may be complemented by diffusion and exchange of components between organelles [45]. Accordingly, transferred mitochondria may fuse with the network of the acceptor cell and thereby “escape” from measurements. Using an *OPA1* KO cell line, which is deficient for mitochondrial fusion, we observed that individual *OPA1* KO cells received larger amounts of mitochondria compared with what we detected in other cell lines (e.g. more transferred mitochondria per fibroblast; see Fig. 3b). However, the overall transfer rate regarding the total cell population was not increased compared with other mouse fibroblast cell lines. This suggests that mitochondrial transfer per cell might be underestimated in fibroblast lines with normal mitochondrial dynamics. In general, the fate of transferred mitochondria remains unclear. Apart from fusion with the mitochondrial network of the acceptor cell, it might also be possible that transferred mitochondria are rapidly degraded via mitophagy, which might in part be responsible for the low detection rates of “mito-positive” fibroblasts. However, the increase of mitochondrial mass in fibroblasts that received mitochondria from MSCs suggests that incorporation into the mitochondrial network of the acceptor cell is the primary mechanism.

In view of the OXPHOS defect, the main question certainly is to what extent cell contacts between MSCs and fibroblasts with mitochondrial transfer are beneficial for cell metabolism. Of note, dysfunction of the OXPHOS system is known to alter mitochondrial ROS production. As depicted in Fig. 4, levels of MitoSOX Red-oxidizing ROS were significantly increased in NDUFS4-deficient cell lines under non-treated conditions. Interestingly, in embryonic mouse fibroblasts, this increase was only detectable when culturing the cells in low-glucose medium, indicating that these cells may compensate the metabolic problem in case of high glucose supply [46]. After co-culture with MSCs, we observed a clear reduction of ROS levels in NDUFS4-deficient fibroblasts that received MSC mitochondria. In human cell lines, ROS levels even normalized completely. However, also in the “mito-negative” cell population, we observed clear effects on ROS levels indicating that the underlying mechanism might not only be related to mitochondrial transfer. This idea was further supported by the observation that treatment with cell-free MSC culture medium supernatant had similar effects on ROS levels.

MSCs are known to contain high levels of glutathione [47]. Glutathione is a substrate for glutathione peroxidase and other antioxidant enzymes, which represents a crucial cellular protection system against oxidative stress [48, 49]. Ayatollahi et al. [49] showed that oxidative stress could be significantly reduced via MSCs in rats with a carbon tetrachloride-induced liver damage. In view of the presented results, it appears highly likely that glutathione and/or other antioxidants are transferred to the NDUFS4-deficient fibroblasts (most likely via multiple mechanisms including TNTs and microvesicles), leading to the reduction in ROS levels. Modulation of cellular redox homeostasis appeared to be specific for MSCs and was not observed after co-culture/treatment of NDUFS4-deficient fibroblasts with healthy control fibroblasts/control medium supernatant. In contrast, mitochondrial transfer was not entirely MSC specific in the human cell model system and was also visible after co-culture of NDUFS4-deficient fibroblasts with control fibroblasts.

Despite the aforementioned effects of MSCs on redox homeostasis, it remains to be answered in how far OXPHOS function can be improved in NDUFS4-deficient fibroblasts via MSC treatment. However, for analysis of mitochondrial CI expression and activity, MSCs and fibroblasts need to be separated after co-culture and a high purity of sorted cells is required. Moreover, relatively large amounts of cells are necessary. We were unable to achieve these experimental conditions in our co-culture system. Based on the observation that treatment of fibroblasts with MSC medium supernatant achieved similar effects on ROS levels compared with co-culture, we proceeded with analysing CI function in this experimental setting. As depicted in Fig. 5, treatment of NDUFS4-deficient fibroblasts with MSC supernatant had no visible effects on levels of fully assembled CI in human and mouse cell lines.

## Conclusions

This study clearly indicates cellular interactions and mitochondrial transfer between MSCs and human as well as mouse fibroblast cell lines. However, mitochondrial transfer appeared to be a relatively rare phenomenon and the transfer rate could only be stimulated moderately by TNF-α treatment. The fate and functional relevance of transferred mitochondria remained elusive. Moreover, mitochondrial transfer in our cell culture systems was not entirely specific for MSCs and was also observed between control and patient-derived fibroblasts. In contrast, MSCs effectively lowered cellular ROS levels in NDUFS4-deficient fibroblast cell lines. This appeared to be a phenomenon that was at least partially independent of mitochondrial transfer and is putatively mediated via secreted factors (e.g. microvesicles). Modulation of cellular redox homeostasis was MSC specific and could not be achieved via control fibroblast cell lines. However, CI expression and activity could not be rescued by treatment with MSC medium supernatant. In view of this observation and the relatively low mitochondrial transfer efficiency during direct co-culture conditions, it appeared unlikely that an MSC-based treatment approach in NDUFS4-related CI deficiency directly enhances cellular energy metabolism. Nevertheless, modulating and mitigating ROS production via MSCs may be an interesting starting point for further in-vivo research studies involving established mouse models of mitochondrial NDUFS4 deficiency.

## Additional files

Additional file 1: (**A**) Western blot analysis from murine *NDUFS4* KO and WT cells and from human patient and human control cells. In *NDUFS4* KO cells and in NDUFS4-deficient patient cells, no NDUFS4 protein expression is detectable. VDAC (voltage-dependent anion channel) used as loading control. (**B**) Biochemical measurements from fibroblasts of the NDUFS4-deficient patient, demonstrating severe CI deficiency. For details regarding methods that were used to measure OXPHOS enzyme activities in fibroblasts [50]. (TIF 92 kb)

Additional file 2: (**A**) Representative fluorescence images showing a mouse fibroblast with LMNB BFP-labelled nucleus, a mouse stem cell (mMSC) with Cox8a GFP-labelled mitochondria and a mouse fibroblast containing mitochondria derived from mMSCs. Cell boundaries are stained with phalloidin. (**B**) Confocal image depicting a mouse fibroblast with Cox8a RFP-labelled mitochondria in co-culture with an mMSC containing Cox8a GFP-labelled mitochondria. (**C**) Representative confocal images of human cells. *Left*: human fibroblasts with Cox8a RFP-labelled mitochondria without transferred mitochondria (*upper image*) or after mitochondrial transfer via hMSCs (*lower image*). *Right*: Cox8a GFP-labelled hMSCs with Cox8a GFP-labelled mitochondria without transferred mitochondria (*upper image*) or after mitochondrial transfer from human fibroblasts (*lower image*). *Scale bar* represents 10 μm. (TIF 912 kb)

Additional file 3: (**A**) Mitochondrial transfer between mouse fibroblasts and mMSCs. Representative fluorescence image of TNTs between fibroblast and mMSC (*white arrow*). *Scale bar* represents 10 μm. (**B**) Representative flow cytometry analysis images for analysing of mitochondrial transfer. Gating procedure of LMNB RFP positive fibroblasts with transferred Cox8a GFP positive MSC mitochondria. *Black arrows* indicate sequential analysis steps. Cells (fibroblasts and MSCs) were selected on the basis of cellular size (forward scatter area, FSC-A) and granularity (side scatter area, SSC-A). Only LMNB RFP positive fibroblasts were used for the next step. Cell doublets were excluded by comparing SSC-H (side scatter height) and SSC-W (side scatter width). Double positive fibroblasts were determined. (TIF 670 kb)

Additional file 4: Is a time-lapse video showing a NDUFS4-deficient mouse fibroblast. Mouse fibroblast mitochondria are labelled (*red*, Cox8a RFP). The fibroblast contains several *green* mitochondria (Cox8a GFP labelled) which are derived from mMSCs. Please note the dynamic motility of mitochondria during the time of video recording. (AVI 1038 kb)

Additional file 5: Is a time-lapse video showing a NDUFS4-deficient human fibroblast. Human fibroblast mitochondria are labelled (*red*, Cox8a RFP). The fibroblast is in contact with a hMSC cell that appears to transfer *green* mitochondria (Cox8a GFP labelled). Please note the dynamic motility of mitochondria during the time of video recording. (AVI 1248 kb)

## Abbreviations

2DG: 2-Deoxy-d-glucose
BN-PAGE: Blue native polyacrylamide gel electrophoresis
BSO: Buthioninesulfoximine
CI: Complex I
Gal: Galactose
GVHD: Graft-versus-host disease
LHON: Leber hereditary optic neuropathy
MELAS: Mitochondrial encephalomyopathy, lactic acidosis and stroke-like episodes
MERRF: Myoclonic epilepsy with ragged red fibre
MSC: Mesenchymal stem cell
NDUFS4: NADH dehydrogenase ubiquinone Fe-S protein 4
OPA1: Optic atrophy 1 (mitochondrial fusion protein)
OXPHOS: Oxidative phosphorylation
ROS: Reactive oxygen species
TNT: Tunnelling nanotube
