# Supplementary figures and images for: Modulation of oxidative phosphorylation and redox homeostasis in mitochondrial NDUFS4 deficiency via mesenchymal stem cells
Source: Stem Cell Res Ther. 2017 Jun 24;8:150. doi: 10.1186/s13287-017-0601-7 (PMC5482938; doi:10.1186/s13287-017-0601-7)

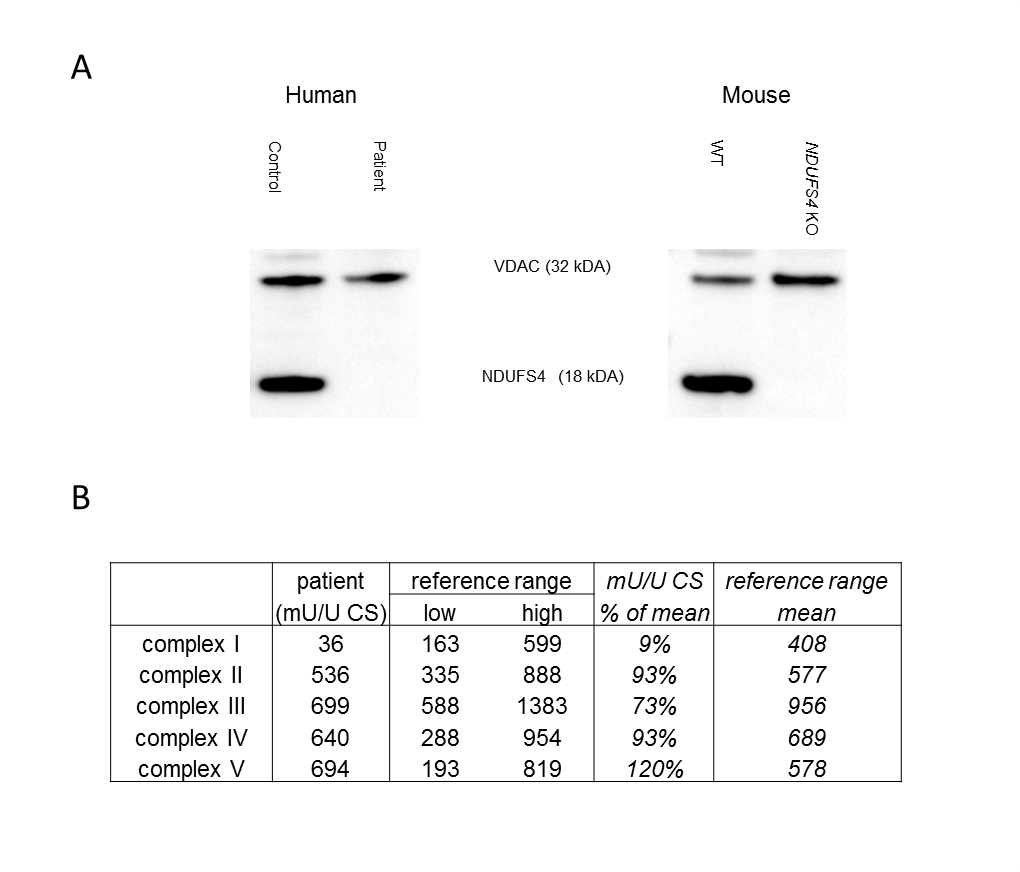

Supplement: Supplementary file 1 — (A) Western blot analysis from murine NDUFS4 KO and WT cells and from human patient and human control cells. In NDUFS4 KO cells and in NDUFS4-deficient patient cells, no NDUFS4 protein expression is detectable. VDAC (voltage-dependent anion channel) used as loading control. (B) Biochemical measurements from fibroblasts of the NDUFS4-deficient patient, demonstrating severe CI deficiency. For details regarding methods that were used to measure OXPHOS enzyme activities in fibroblasts [50]. (TIF 92 kb) [file 13287_2017_601_MOESM1_ESM.tif]

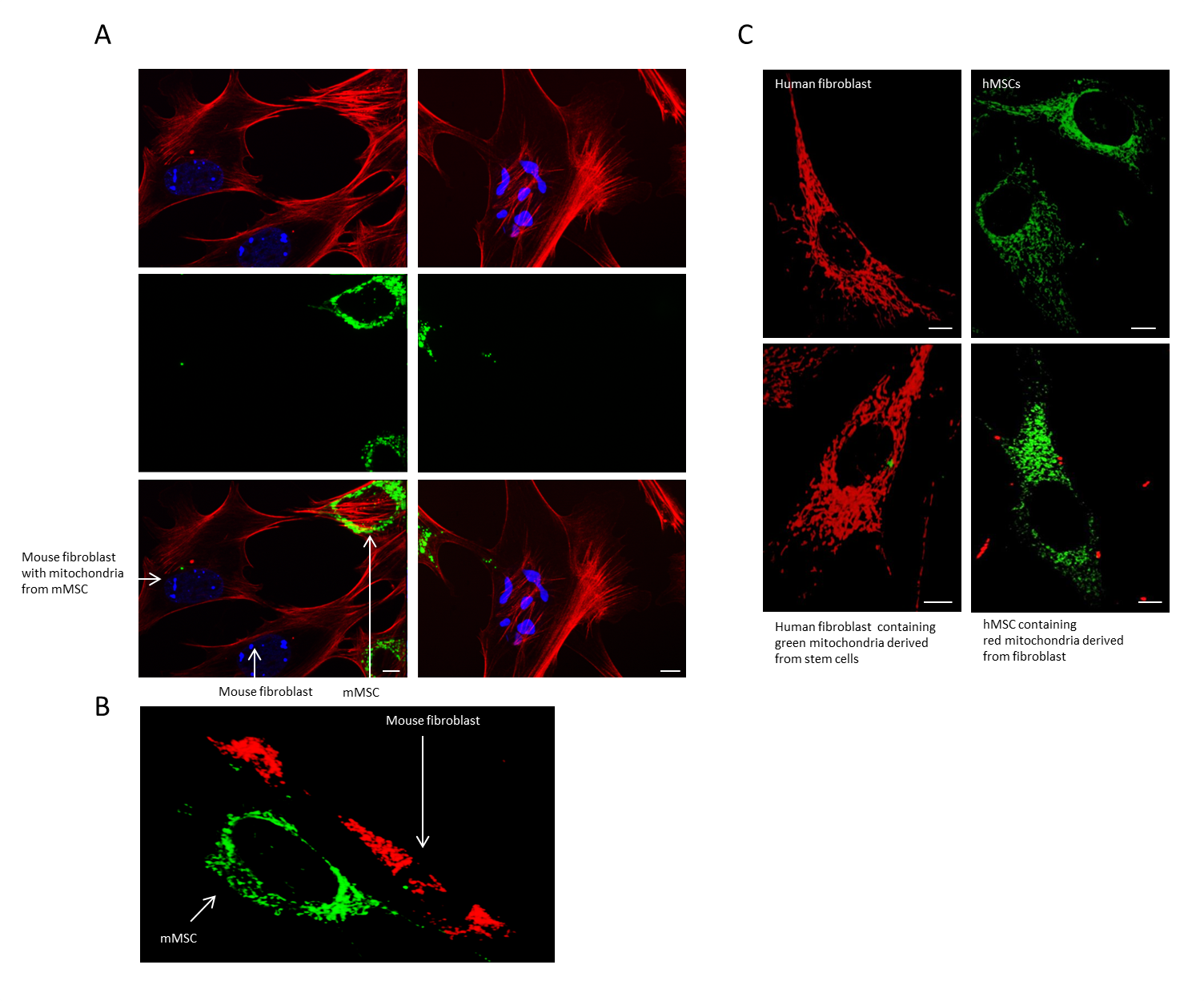

Supplement: Supplementary file 2 — (A) Representative fluorescence images showing a mouse fibroblast with LMNB BFP-labelled nucleus, a mouse stem cell (mMSC) with Cox8a GFP-labelled mitochondria and a mouse fibroblast containing mitochondria derived from mMSCs. Cell boundaries are stained with phalloidin. (B) Confocal image depicting a mouse fibroblast with Cox8a RFP-labelled mitochondria in co-culture with an mMSC containing Cox8a GFP-labelled mitochondria. (C) Representative confocal images of human cells. Left: human fibroblasts with Cox8a RFP-labelled mitochondria without transferred mitochondria (upper image) or after mitochondrial transfer via hMSCs (lower image). Right: Cox8a GFP-labelled hMSCs with Cox8a GFP-labelled mitochondria without transferred mitochondria (upper image) or after mitochondrial transfer from human fibroblasts (lower image). Scale bar represents 10 μm. (TIF 912 kb) [file 13287_2017_601_MOESM2_ESM.tif]

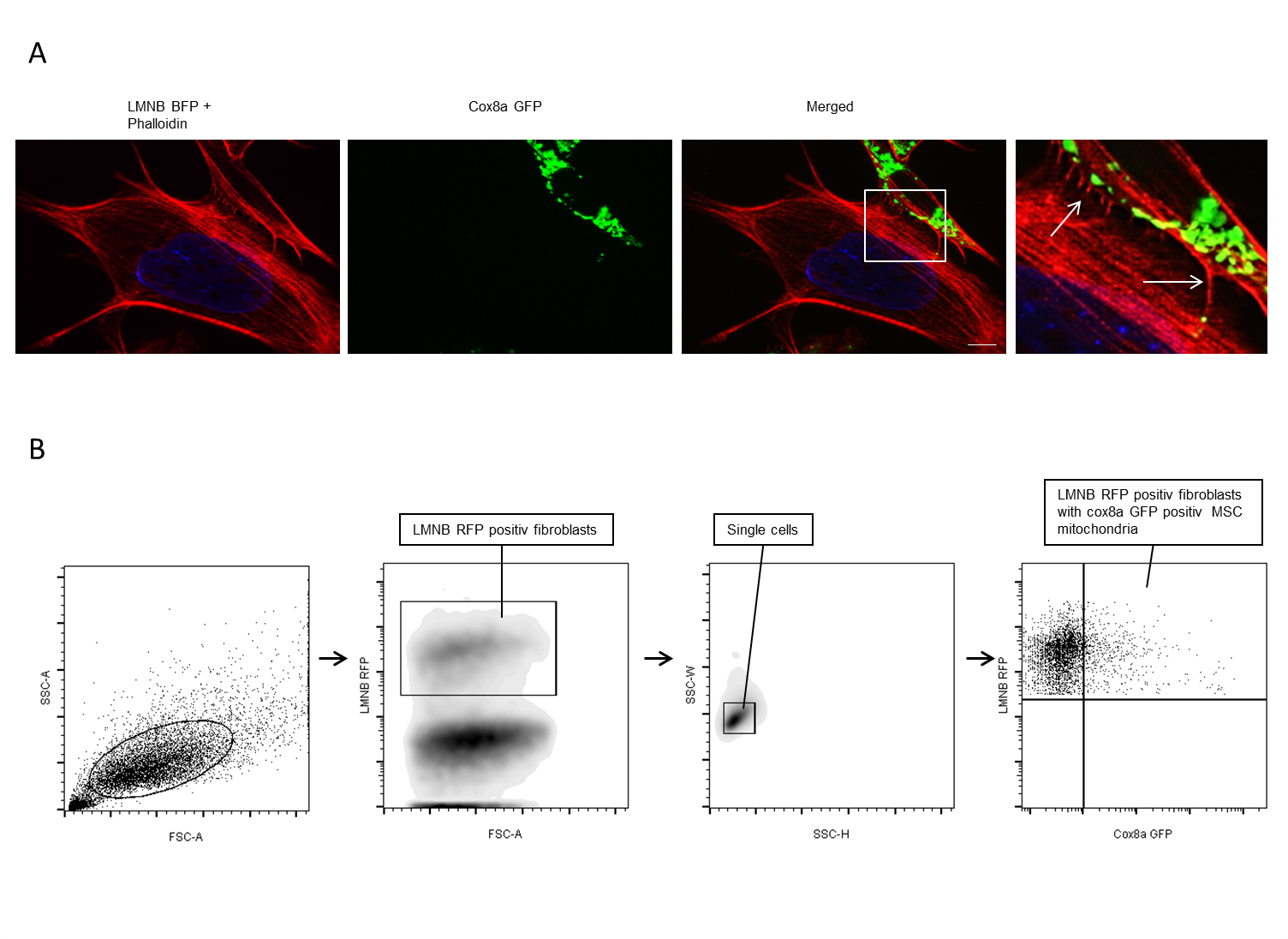

Supplement: Supplementary file 3 — (A) Mitochondrial transfer between mouse fibroblasts and mMSCs. Representative fluorescence image of TNTs between fibroblast and mMSC (white arrow). Scale bar represents 10 μm. (B) Representative flow cytometry analysis images for analysing of mitochondrial transfer. Gating procedure of LMNB RFP positive fibroblasts with transferred Cox8a GFP positive MSC mitochondria. Black arrows indicate sequential analysis steps. Cells (fibroblasts and MSCs) were selected on the basis of cellular size (forward scatter area, FSC-A) and granularity (side scatter area, SSC-A). Only LMNB RFP positive fibroblasts were used for the next step. Cell doublets were excluded by comparing SSC-H (side scatter height) and SSC-W (side scatter width). Double positive fibroblasts were determined. (TIF 670 kb) [file 13287_2017_601_MOESM3_ESM.tif]
